# Should there be a World Health Assembly resolution for malaria eradication? Opinion against

**DOI:** 10.1186/s12936-019-2928-2

**Published:** 2019-10-21

**Authors:** T. Jacob John

**Affiliations:** 1 Departments of Clinical Microbiology and Clinical Virology, Christian Medical College, Vellore, Tamil Nadu India; 2 Thekkekara, 439 Civil Supplies Godown Street, Kamalakshipuram, Vellore, Tamil Nadu 632002 India

## Abstract

A resolution for eradicating malaria, if passed by the World Health Assembly (WHA), will have a distracting effect on all countries with malaria. The continued prevalence of malaria is indicative of weak public health infrastructure. True, smallpox was eradicated by international efforts following WHA resolution: the success factor was primary prevention using a safe and effective vaccine. A resolution to eradicate polio was passed in 1988, with a target year of 2000, but even in 2019 success is not within reach. Public health experts are hesitant to move forward with measles eradication before polio is eradicated. Country by country elimination of malaria is a better way, ensuring the strengthening of public health infrastructure, with many other health benefits.

## Magnitude of malaria

An estimated 219 million malaria cases with 435,000 deaths occurred in 2017 in 87 countries—92% to 93% in WHO Africa Region [1]. Other malarial countries are spread in all other WHO Regions except European [1]. Imagine how great it would be if all cases and deaths could be averted through malaria eradication. Will a World Health Assembly (WHA) Resolution expedite eradication? A strongly worded resolution will ease the consciences of some global health leaders. Are malarial countries ready for applying the necessary interventions?

Disease control hierarchy is: control, elimination (of disease, of infection), eradication, extinction [2]. Eradication is achieving zero incidence, worldwide, of human infection with *Plasmodium falciparum, Plasmodium vivax, Plasmodium ovale* and *Plasmodium malariae* [2]. Female *Anopheles* mosquitoes and humans are their definitive and intermediate hosts, respectively. Eradication requires both hosts rendered infection-free. For now, zoonotic malaria will be ignored.

The life span of the vector is < 3 weeks; ‘extrinsic incubation period’ takes 8–10 days. So, for each mosquito, the interval to infect humans is very short. Therefore, to sustain endemic malaria, new mosquitoes must be continuously infected. This sounds like precarious existence for malaria. In reality, malaria is tenacious where a combination of prolific vector breeding and human inability to prevent their repeated blood meals co-exists.

## Tension between two approaches

Tension between ‘health for all’ (HFA) and ‘selective disease control’ (SDC) approaches for disease control has existed for > 4 decades. HFA envisages the establishment of universal healthcare to diagnose and treat sicknesses promptly and of public health to mitigate environmental and social risk factors. Together they reduce the frequency of infection in humans and vectors, resulting in malaria control. If sustained, the pathogen tends to ‘die out’ as the disease’s ‘reproduction rate’ R falls below 1.

Socio-economic development leads to an intolerance of infectious diseases; developed nations achieved malaria control and elimination through HFA approach. Global health leaders have not designed a blueprint to create universal healthcare and public health in countries that need them most urgently. Without them malaria elimination is virtually impossible. For many poor countries, development, disappointingly, is too slow a path to eliminate malaria. Therefore the alternate SDC approach—eradicate the pathogen—is attractive to many.

In the first half of twentieth century, thus, two conceptual camps arose: one favouring large-scale campaigns of vector control, aiming for rapid malaria eradication and the other favouring locally designed, progressive, albeit slow, development of healthcare and environmental sanitation, to progressively reduce malaria morbidity and mortality [3].

Dichloro-diphenyl-trichloroethane (DDT) as a highly effective indoor residual insecticide became a game-changer of vector-control in early 1940s. Its promise led to the WHA resolution for Global Malaria Eradication in 1955 [3]. A Global Malaria Eradication Programme (GMEP) was established for “*ending of transmission of malaria and the elimination of the reservoir of infective cases in a campaign limited in time and carried out to such a degree of perfection that when it comes to an end, there is no resumption of transmission*” [3]. The strategy was to spray DDT systematically with monitoring of malaria with set criteria. Sustained malaria diagnosis and treatment were not possible without universal primary healthcare.

Soon DDT became a popular agricultural pesticide and mosquitos developed increasing resistance [3]. Within a decade GMEP was fatigued even while a quarter of malarial regions had not come under eradication interventions [3]. Then in 1968–69, Sri Lanka had epidemic resurgence of malaria after excellent control, almost to the point of elimination [3]. India and many other countries also experienced massive resurgence and shifting epidemiology—urban malaria, previously rare, became very common and vexatious. In 1969, the WHA admitted failure, stating: “*In the regions where eradication does not yet seem feasible, control of malaria with the means available should be encouraged and may be regarded as a necessary and valid step towards the ultimate goal of eradication*” [3]. Unfortunately, by then, the earlier, slowly built-up malaria control foundations of country-based, locally experienced, malaria experts had been disbanded; consequently malaria control became extremely difficult in many countries that had participated in eradication plan [3].

## Success and failures of other eradication programmes

Smallpox eradication is the only success story of a WHA resolution (passed in 1959). The last case of community-acquired smallpox was in 1976; eradication was certified in 1980. It proved the power of vaccines to eradicate diseases without extra-human reservoir—such as polio and measles. In 1988, WHA passed a resolution to eradicate polio by 2000. Experts seemed to have learned wrong lessons from smallpox eradication.

With smallpox success in 16 years, for polio 12 years were considered sufficient. Smallpox was eradicated using live virus vaccine; so, for polio the live oral poliovirus virus vaccine (OPV) was used exclusively, ignoring the potential of inactivated poliovirus vaccine (IPV). The lessons from malaria—that it was unwise to use only one intervention tool universally, or make one size fit all, were already forgotten. Consequently the 12-year sprint of polio eradication has now become 31-year-and-still-running marathon. Moreover, the moral dilemma of many times more OPV-caused polio than natural polio is haunting the programme. The general frustration among many public health opinion leaders was succinctly put: “[No] *measles eradication resolution is likely until member states are satisfied that polio eradication is accomplished*” [4].

Removing smallpox virus without addressing the disparities of health management systems in countries was ‘excision surgery’. It worked because the disease and its vaccine were unique. It provides only proof of principle, not a model for replication.

Any WHA resolution to eradicate a disease must not be taken until after designing and validating intervention measures, under a common strategy but flexible tactics. Interventions must be based on robust science and public health ethics. Without such preparation a resolution will pressurise countries, public health personnel and WHO itself, to pick one technological tool and to make one size fit all, losing sight of the complexity of the target disease’s epidemiology and the state of the health management systems of the countries needing extra external help.

## There are no short-cuts

Scientific medicine consists of public health (to prevent preventable diseases), universal healthcare (to treat what was not prevented) and research to constantly raise the bars of both. The philosophical principle of social justice—that people’s health is State’s responsibility—became the political ideology in democracies that desired human development. When scientific medicine was transplanted into cultures familiar only with various traditional medicines, the three elements did not take roots. Instead, therapeutics and surgery are eagerly accepted but public health and universal healthcare are neglected.

The Expanded Programme on Immunization (EPI) was designed for countries without public health and universal healthcare. It should have been the entry point to design public health and universal healthcare in all countries, with practical disease surveillance and self-reliant disease control [5]. The spinoff benefit, to cite one example, would have been the control of tuberculosis (TB), rightly declared a global emergency in 1993 but not matched with a strategy to face the emergency. Like EPI and TB control that still remain ‘vertical’, every eradication programme by WHA resolution will also be ‘vertical’.

To think outside the box and design health management systems in countries that vary widely in their skills, capacities and funds, is the need of the hour. Every WHO Region has an office and technically skilled personnel. How to energize them to interact with each member country is the challenge. The America Regional Office (PAHO) functions this way and serves as a model for others.

There are no short cuts to development and disease control. Health is not only a measure of, but also a means to development. Investments in health will result in huge dividends—convincing countries and getting their endorsement and ownership are essential pre-requisites to eventual malaria eradication through country-by-country control and elimination.

Countries that have autonomously designed and achieved malaria elimination show us the way forward: Sri Lanka was declared malaria eliminated in 2016 and Algeria and Argentina in 2019. It is never too late to enable and empower countries to control all important infectious diseases. An eradication resolution for malaria eradication will simply be a distractor.

## Data Availability

Not applicable.
